# Understanding barriers, enablers and motivational factors for Australian healthcare educators teaching university students on clinical placement using the validated Physician Teaching Motivation Questionnaire

**DOI:** 10.1186/s12909-024-05886-1

**Published:** 2024-08-21

**Authors:** Natalie Ann Watt, Simon Backhouse, Saba Ansari, Karen Maree Dwyer, Janet McLeod, Grant Phelps, Deborah Leach, James Andrew Armitage

**Affiliations:** 1 School of Medicine, Faculty of Health, Deakin University, 75 Pigdons Road, Waurn Ponds, 3216 Australia; 2 School of Medicine (Optometry), Faculty of Health, Deakin University, 75 Pigdons Road, Waurn Ponds, 3216 Australia; 3 School of Medicine (Medical Imaging), Faculty of Health, Deakin University, 75 Pigdons Road, Waurn Ponds, 3216 Australia

**Keywords:** Teaching motivation, External healthcare professionals, External healthcare educators, Validated questionnaire, Teaching benefits and barriers

## Abstract

**Background:**

In Australia, university clinical programs rely heavily on external healthcare professionals to provide a range of authentic clinical training and professional development opportunities for students. There is, however, a limited understanding of the factors that motivate these professionals to be educators and whether this varies across different healthcare disciplines within Australia. As the demand for clinical placements continues to increase, it is critical for the ongoing success of these programs that universities identify both the barriers associated with teaching and the benefits that enhance job satisfaction.

**Methods:**

A two-part questionnaire was emailed to 849 external healthcare educators teaching predominantly in Medicine, Medical Imaging, and Optometry programs at Deakin University. Part-one surveyed the socio-demographics, perceived benefits, and barriers to teaching, plus the utilisation of entitlements offered by the university. Part-two was modelled on Dybowski and Harendza’s validated Physician Teaching Motivation Questionnaire (PTMQ) and adapted to an Australian audience.

**Results:**

Overall, 776 out of the 849 questionnaires were considered in the final participant pool. The response rate for part-one was 19.7% (*n* = 153/776) and 18.3% (*n* = 142/776) for part-two. Across all professions, altruism was the primary source of motivation for teaching in Deakin University’s healthcare programs. Medical Imaging educators placed a higher value on career-related teaching aspects compared to those in Medicine and Optometry (*p* < 0.003). The resources and benefits associated with teaching were generally perceived to be of minimal importance. External healthcare educators most commonly reported that there were no barriers to teaching (41.3%, *n* = 78) however, increased workloads and time constraints were the next most likely factors to affect teaching participation (31.8%, *n* = 60).

**Conclusion:**

Our validated adaptation of the PTMQ successfully targeted healthcare professions not focussed on by Dybowski and Harendza. Altruistic motivation was the overarching theme for why Australian external healthcare educators contribute to teaching with some differences in career-driven motivation additionally acknowledged. Despite there being no key benefits or barriers to teaching participation, universities must remain cognisant of the factors that influence the recruitment and retention of external healthcare educators and how to optimise these for the ongoing success and sustainability of their teaching programs.

**Supplementary Information:**

The online version contains supplementary material available at 10.1186/s12909-024-05886-1.

## Background

An authentic clinical experience is the centrepiece of all modern healthcare programs. To fulfil this requirement, medical education is increasingly moving away from traditional hospital-based learning and other institutionalised settings to private or community-based healthcare practices [1–4]. As such, external healthcare professionals who are acting as educators (hereafter referred to as external healthcare educators), play a pivotal role in the clinical training and professional development of healthcare students [3, 4].

As teaching models evolve and the number of medical and allied health students continues to rise, there is a growing demand for clinical placements. To ensure healthcare programs remain sustainable and successful, it is essential to better understand and cultivate motivating factors that encourage external healthcare educators to engage in teaching. Furthermore, given the primary loyalty of external healthcare educators is to their own clinical practice, gaining a clearer insight into the benefits and barriers associated with teaching may help universities develop strategies that optimise or mitigate these factors. This knowledge has the potential to further strengthen initiatives aimed at improving the recruitment and retention of external healthcare educators teaching in university curriculums.

Over the past decade, several international studies have increased our understanding of the drivers behind clinical teaching participation, with selflessness and altruism emerging as the primary motivators [5–8]. Nonetheless, it is important to acknowledge that variations in motivational factors may exist across countries and professions due to cultural diversity, differing training pathways, and teaching styles [9, 10]. To the best of our knowledge, clinical teacher motivation in Australia is limited to only a few studies that predominantly focus on the experience of medical educators in hospital settings [10–15]. Although the findings of these studies are consistent with the aforementioned international findings of altruism, none of them used a validated questionnaire to compare and contrast motivational factors of external healthcare educators across a range of healthcare professions, especially those primarily based in private or community practice settings where students are increasingly being placed.

Dybowski and Harendza [16] originally developed the Physician Teaching Motivation Questionnaire (PTMQ) for hospital-based healthcare practitioners in Germany but suggested that there was a need to validate its generalisability to other allied health professions. With this recognised limitation and the paucity of literature on teacher motivations among Australian healthcare educators, the PTMQ was applied across the three largest clinical courses taught within Deakin University‘s School of Medicine: Bachelor of Vision Science/ Master of Optometry, Doctor of Medicine, and Bachelor of Medical Imaging (diagnostic radiographers).

The success of these three professional degrees largely relies on experienced external healthcare educators teaching and passing on their knowledge to future healthcare graduates. While some of these external healthcare educators have formal educational qualifications and paid fractional appointments, many undertake this work on a casual or adjunct (unpaid members of the university community) basis. Similar to other universities, Deakin University also provides various benefits to their external healthcare educators to increase the value of their teaching contributions including, but not limited to, email accounts, access to university libraries, and research assistance. It is therefore incumbent on universities, including Deakin, to regularly evaluate the benefits and barriers associated with teaching to maintain maximum satisfaction and long-term engagement with external healthcare educators. Therefore, the aims of this study were to:

1. Understand what motivates external healthcare educators to contribute to university teaching programs.
2. Understand why they maintain their relationship with Deakin University.
3. Identify any perceived barriers associated with healthcare teaching.
4. Identify which benefits external healthcare educators value during their affiliation with the university.

## Methods

### Study design and sampling

This study utilised a cross-sectional survey design, selected for its ability to examine a representative cross-section of the population and generate findings that could be generalised to the entire target population. The total population of healthcare educators registered to supervise Medicine, Medical Imaging or Optometry students from Deakin University were invited to participate via email.

### Participants

A total of 849 external healthcare educators, defined in this study as staff appointed to undertake teaching and/or research but whose primary employment is with an organisation external to Deakin University, were invited to participate in an online questionnaire-based survey sent via email from a senior administrative officer in October 2020. All participants primarily taught within Deakin University’s School of Medicine. The email addresses used consisted of a combination of personal and university-provided accounts. Of these 849 participants, 273 were from Medicine, 158 from Medical Imaging, and 418 from Optometry.

### Questionnaire

The Qualtrics questionnaire was divided into two parts. Part-one (26 non-forced choice questions), evaluated participants’ socio-demographics, perceived barriers to healthcare teaching, and perceptions of benefits valued and used by external healthcare educators (see Additional file 1). Participants were asked to rate their usage and perceived value of the resources and benefits available to them at Deakin University using a 5-point Likert scale. The 5-point Likert scale used for perceived value was 1 = Extremely important, 2 = Very important, 3 = Moderately important, 4 = Slightly important and 5 = Not at all important and for usage, 1 = Daily, 2 = Weekly, 3 = A few times a month, 4 = A few times a year and 5 = Never.

For perceived barriers associated with teaching, participants were able to select more than one barrier. Each barrier was assigned a number to assist in the analysis of the results (no barriers = 1, the requirements of the HR onboarding process = 2, the requirements of ongoing compliance = 3, competing work requirements/time management = 4, income expectations = 5, other = 6 and IT requirements = 7).

Part-two (18 forced-choice items), was based on Dybowski and Harendza’s [16] PTMQ, a validated multidimensional self-assessment instrument developed to measure teaching motivation within the German medical system. This 18-item questionnaire was grouped into the same six motivational subdomains as the PTMQ: Intrinsic (items 1–4; indicating that they are intrinsically motivated to teach), Identified (items 5–7; professional identity is a motivator), Introjected (items 8–9; motivation is driven by guilt or a sense of duty), Career (items 10–12; there are clear benefits to career progression), External (items 13–15; motivation is driven by a desire to comply with the expectation of others), and Amotivation (items 16–18; teaching is viewed in a negative context). Part-two could only be accessed if part-one was completed.

As the PTMQ survey was developed for the German medical system, the phrasing of some subdomain items was carefully modified to suit Australian language conventions (see Additional file 1). Modifications were made to all items except for 2, 11 and 13. Predominantly, one-word synonym changes were made to avoid affecting the validity of the questionnaire. For example, the original PTMQ item 1 ‘ I look forward to my next teaching unit most of the time’, was revised to ‘I look forward to my next teaching *session* most of the time’. In the ‘Career’ sub-domain, changes were made to stay in line with modern Australian industrial relations terminology. For example, the original PTMQ item 10, ‘I teach because I need the lessons to accomplish my occupational objectives’ was adapted to ‘I teach because *it is good for my CV* to accomplish my occupational objectives’. The modifications were undertaken by the first and last authors and then circulated to the remaining researchers for review. All researchers agreed on the changes. Language experts were not consulted during this process.

As per the PTMQ, a 5-point Likert scale was used to rate each item (1 = Strongly agree, 2 = Somewhat agree, 3 = Neither agree nor disagree, 4 = Somewhat disagree, 5 = Strongly disagree).

The final version of questionnaire part-one and two were piloted on a selection of healthcare clinicians and educators across Medicine (*n* = 3), Medical Imaging (*n* = 1), and Optometry (*n* = 3) employed at Deakin University to confirm general understandability. These individuals were not included in the participant pool. Following the pilot test, no additional changes were made to the final version of the questionnaire as part-one achieved good face validity based on oral and written feedback from the pilot group.

### Data collection

An anonymous link to a Qualtrics questionnaire (a web-based software program version October 2020, Qualtrics, Provo, UT. https://www.qualtrics.com) was provided in the email. A second round of reminder emails were sent six weeks later. The survey ran for 12 weeks from October to December 2020.

### Data preparation

All data from parts one and two of the questionnaire was exported from Qualtrics for statistical analysis using IBM SPSS Statistics for Windows (Version 26.0. IBM Corp., Armonk, NY). All Likert scales were treated as interval scales. Participants who completed less than 10% of the survey were excluded from the statistical analysis. The Kolmogorov – Smirnov test was applied to the data from part-one and part-two to check for normality which confirmed that the data was non-normal.

### Data analysis

#### Questionnaire part-one

Descriptive statistics were generated within SPSS to obtain frequency and percentage responses relating to sociodemographic questions.

#### Questionnaire part-two

As the PTMQ questionnaire was altered to accommodate the Australian language, reliability, and validation analyses were performed using SPSS. A Cronbach’s alpha (α) was applied to each motivational subdomain with α > 0.7 considered to represent an acceptable level, α > 0.8 a good level, and α > 0.9 an excellent level of internal consistency [18].

To align with Dybowski and Harendza’s [16] methodology, a classical factor analysis was conducted to establish construct validity. The Rasch measurement model for polytomous responses, using the Rasch Unidimensional Measurement Model (RUMM) 2030 software package [19], was also used to assess the validity of the PTMQ, looking at unidimensionality and item responses in the full 18-item survey and the identified subdomains [20, 21].

A descriptive-analytic strategy was adopted to summarize the PTMQ item responses by percentage, frequency, median, skewness, and kurtosis. A univariate general linear model with Bonferroni correction for multiple comparisons was used to compare the subdomain and item responses from Medicine, Optometry and Medical Imaging external healthcare educators, with significance set at *p* < 0.05.

### Ethical considerations

This study adhered to the tenets of the Declaration of Helsinki. Ethics approval was granted by Deakin University’s Faculty of Health Human Ethics Advisory Group (HEAG_H 184_2020).

Completion of the survey indicated that participants had read the Plain Language Statement and provided consent to participate. Participants were instructed to close the survey and not proceed if they did not wish to provide consent [17]. By using an anonymous link, no identifiable information was collected. A unique response ID was automatically created for each participant once the Qualtrics questionnaire was completed.

## Results

### Response characteristics (Questionnaire part-one and two)

Of the 849 questionnaires emailed, 73 emails were undeliverable resulting in 776 potential participants. Of these 776 potential participants, 153 completed part-one and 142 completed part-two. Fifteen participants (8.9%) completed fewer than 10% of the questions and were excluded from the analysis giving an overall completed response rate of 19.7% (*n* = 153/776) for part-one and 18.3% (142/776) for part-two. The response rates across each profession for part-one were as follows: Medicine (37.9%, *n* = 58/153), Medical Imaging (10.5%, *n* = 16/153), and Optometry (51.6%, *n* = 79/153). For part-two (PTMQ): Medicine (38.7%, *n* = 55/142), Medical Imaging (11.3%, *n* = 16/142), and Optometry (50.0%, *n* = 71/142). Due to these response variations, and several part-one questions allowing for multiple options to be chosen, percentage responses are quoted along with a fraction, in brackets, representing the total number of participants for that particular item.

### Questionnaire part-one results

#### Participant socio-demographics

Table 1 displays the frequency and percentage distribution of participants’ socio-demographic characteristics. The median age was between 40 and 49 years with more male participants than female. Nearly half of the participants had a Bachelor’s degree as their highest educational qualification, and close to 60% (*n* = 89/153) reported a length of service between one and five years. Half of the participants were involved in teaching Optometry, just over one-third taught in Medicine, and 10% taught in Medical Imaging. The ‘Other’ category included external healthcare educators who were also involved in teaching programs outside of the School of Medicine’s three largest clinical courses such as Paramedicine, Higher Degree Research, and Biomedicine. External healthcare educators’ primary teaching activity was the supervision of students providing clinical care (38.3%, *n* = 106/277). A majority (60.1%, *n* = 92/153) did not have a paid appointment with Deakin University however, all participants (*n* = 153), held an academic appointment title.

**Table 1:** Participant socio-demographics

Participant Socio-demographics	Percentage (*n*)
**Age (yrs)**	
18–29	5.2% (8)
30–39	18.3% (28)
40–49	31.4% (48)
50–59	27.5% (42)
60–69	17.0% (26)
≥ 70	0.7% (1)
**Sex**	
Male	60.8% (93)
Female	39.2% (60)
**Highest Qualification**	
Bachelors	44.4% (68)
Post Grad Degree	24.2% (37)
Masters	24.2% (37)
PhD	7.2% (11)
**Length of Service (yrs)**	
< 1	9.8% (15)
1–5	58.2% (89)
5–10	22.2% (34)
> 10	9.8% (15)
**Course(s) Taught**	
Medicine	36.7% (58)
Medical Imaging	10.1% (16)
Optometry	50.6% (80)
Other	2.5% (4)
**Teaching Activities**	
Delivering lectures	10.5% (29)
Delivering tutorials	22.0% (61)
Clinical Skills Teacher	28.5% (79)
Supervising students providing clinical care	38.3% (106)
Research Training	0.7% (2)
**Paid Appointment**	
Yes	39.9% (61)
No	60.1% (92)

Categories ‘courses taught’ and ‘teaching activities’ have total numbers greater than 153 as multiple options could be chosen.

### Benefits of teaching

**Fig. 1:**
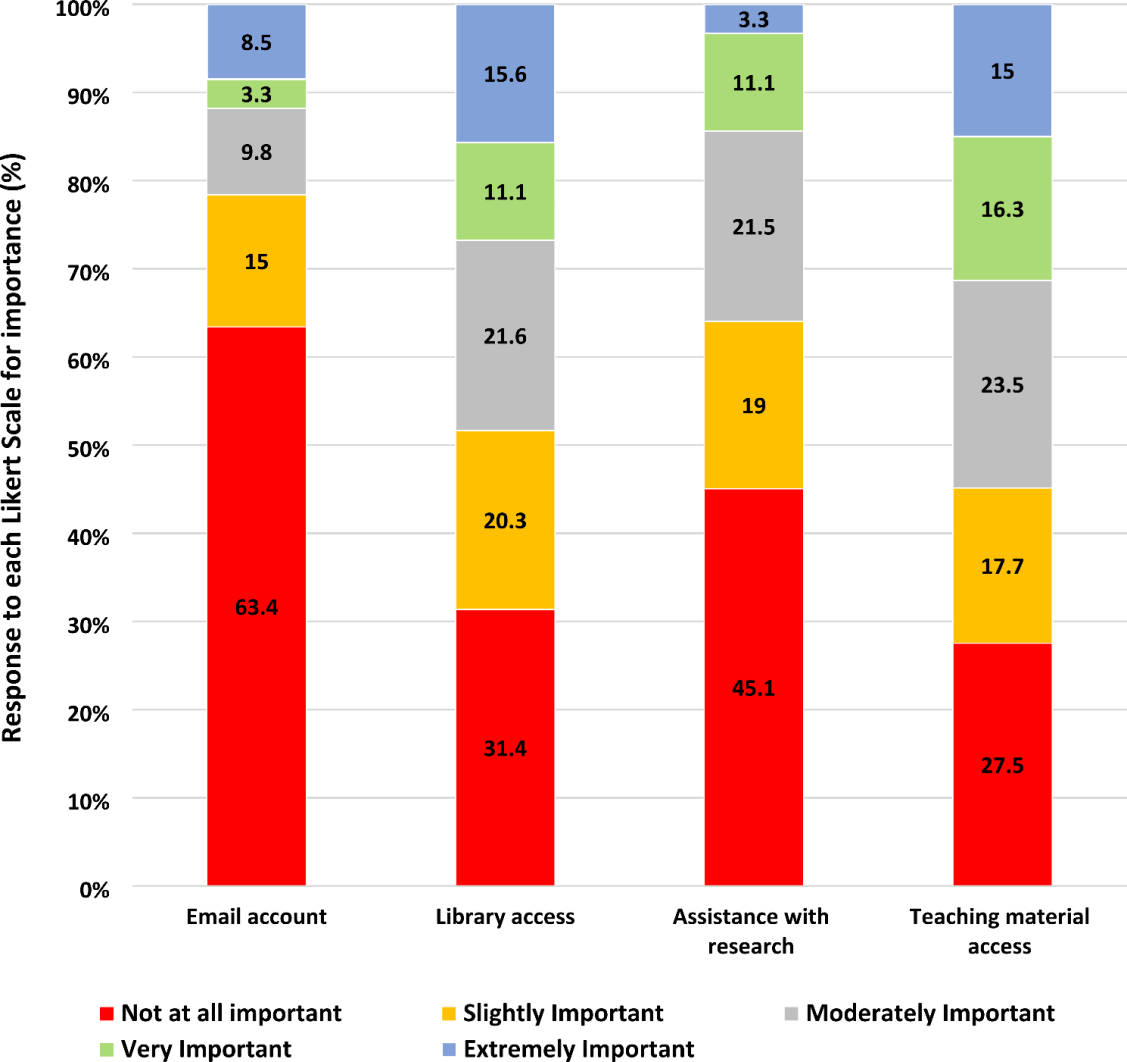
Perceived usefulness of benefits and resources available to external healthcare educators

Across all three professions, the most common response on the perceived value of the resources and benefits associated with teaching at Deakin University was ‘not at all important’ (Fig. 1). Having a university email account was the least valued, with almost half reporting that they did not use their account (46.4%, *n* = 71/153). Although library access was considered slightly more valuable than having an email account, 54.9% (*n* = 84/153) reported having never accessed the library. While most commonly, university assistance with research was not considered important, there was some perceived value in having access to students’ online teaching resources even though only 49.7% (*n* = 76/153) utilised this material.

Continuing professional development, an additional benefit provided by Deakin University, was accessed by 34.6% (*n* = 53/153) of participants, with 27.5% (*n* = 42/153) of those completing only 1–5 h per year.

### Barriers to teaching

**Fig. 2:**
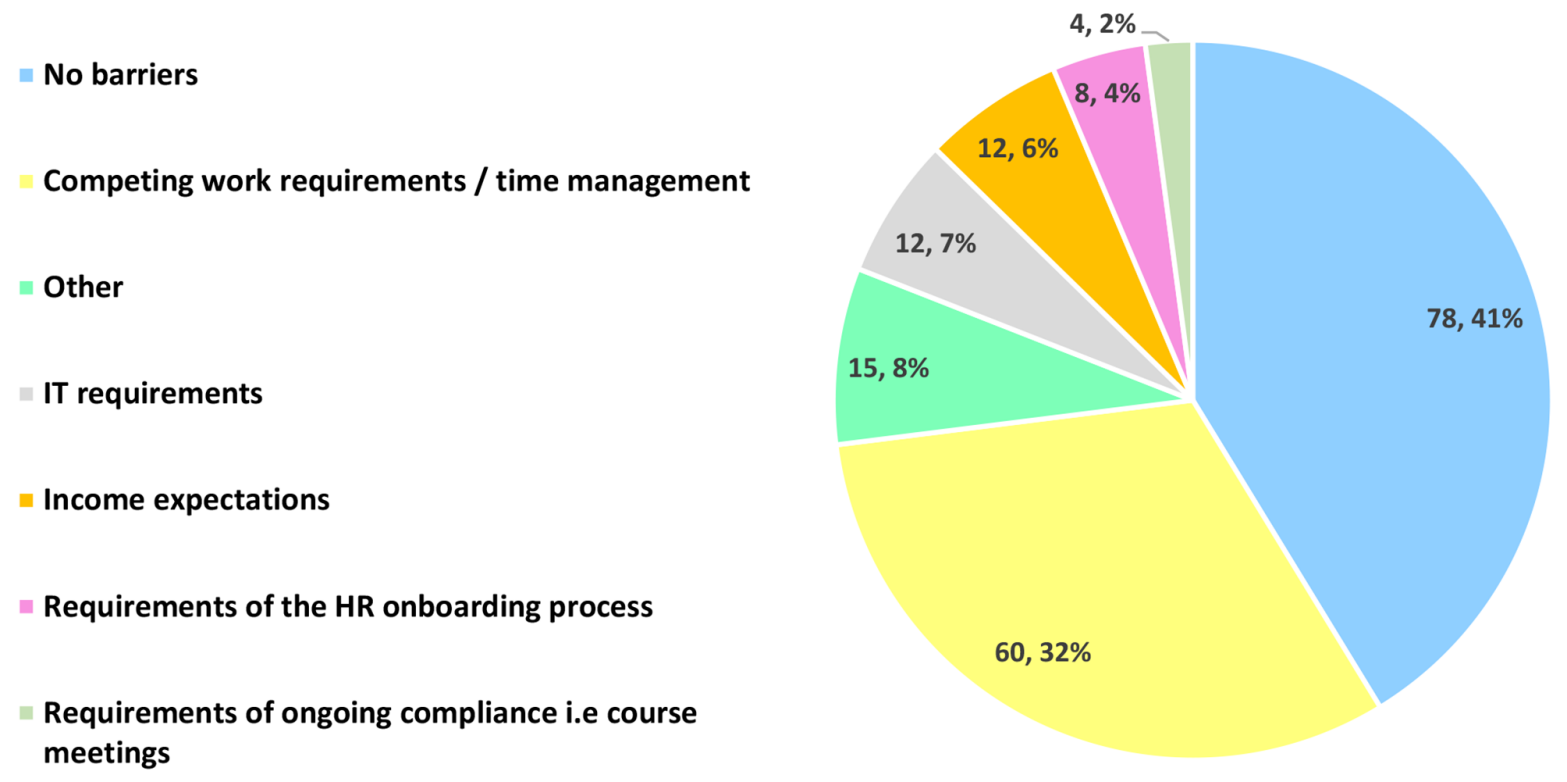
Barriers to teaching

Participants were given the option to choose multiple barriers, therefore, the sum total number of responses is greater than *n* = 153. Figure 2 shows most commonly (41.3%, *n* = 78/189), that there were no barriers to participants maintaining their association with Deakin University. However, 31.8% (*n* = 60/189) felt that competing work requirements and time constraints were potential deterrents. Information and technology (IT) requirements, income expectations, requirements of the onboarding human resources (HR) process, and ongoing university compliance were considered relatively minor barriers. The ‘other’ barrier category included factors such as the location of their practice, an inability to obtain student evaluation feedback, and difficulties with upholding regular communication with the university.

The primary barrier reported by Medicine were competing work requirements and time constraints (46.6%, *n* = 27/58), while the majority of participants in Optometry (58.2%, *n* = 46/79) and Medical Imaging (62.5%, *n* = 10/16), reported that there were no barriers in being able to teach.

### Questionnaire part-two results

#### PTMQ survey items

The final adapted 18 PTMQ survey items, along with the corresponding median, interquartile range, skewness, and kurtosis values for all participants are displayed in Table 2. Overall, the subdomain median values were lowest for Identified and highest for Amotivation. A low median value indicated a ‘strongly agree’ response and a high median indicated a ‘strongly disagree’ response.

**Table 2:** PTMQ items, medians, interquartile ranges, skewness, and kurtosis for all participants

Subdomains	Survey Item	Median (IQR)	Skewness	Kurtosis
**Intrinsic**				
1	I look forward to my next teaching session most of the time	2.00 (1.00)	0.87	0.26
2	I enjoy my teaching most of the time	1.00 (1.00)	1.26	1.63
3	I am completely in my element when teaching	2.00 (1.00)	0.77	0.60
4	I teach because it increases my job satisfaction	1.00 (1.00)	1.51	2.73
**Identified**				
5	I teach because it’s important for me to make a contribution to students becoming a good healthcare professional in the future	1.00 (1.00)	1.62	1.61
6	I teach because I am convinced it’s my duty to pass on my knowledge	2.00 (1.00)	0.99	0.92
7	I teach because I feel that the knowledge I impart is important	1.00 (1.00)	0.72	-0.50
**Introjected**				
8	I teach because otherwise I feel guilty for not helping my colleagues	4.00 (2.00)	-0.38	-0.87
9	I teach because otherwise I feel guilty for not helping my supervisors	4.00 (2.00)	-0.49	-0.66
**Career**				
10	I teach because it is good for my CV to accomplish my occupational objectives	3.00 (2.00)	-0.25	-1.01
11	I teach because it is advantageous to my occupation	3.00 (2.00)	0.45	-0.81
12	I teach because it is good for my career progression	3.00 (2.00)	-0.05	-1.0.
**External**				
13	I teach most of the time because my supervisors expect it from me	4.00 (2.00)	-0.81	-0.37
14	I mainly teach because it is part of my position description	4.00 (2.00)	-0.76	-0.49
15	I mainly teach because otherwise I could be performance managed	5.00 (1.00)	-1.59	2.24
**Amotivation**				
16	I teach even though I feel that teaching is a lower priority than my other occupational activities	4.00 (2.75)	-0.51	-1.04
17	I rarely feel like teaching but do it anyway	5.00 (1.00)	-1.67	2.67
18	I teach even though I often perceive it as an annoying chore	5.00 (1.00)	-1.49	1.48

5 Likert response range: 1 = Strongly agree, 2 = Somewhat agree, 3 = Neither agree nor disagree, 4 = Somewhat disagree, 5 = Strongly disagree.

### PTMQ descriptive responses

Overall, the ‘Identified’ subdomain, comprising items 5, 6, and 7, showed the highest level of congruence, with nearly 60% expressing a ‘strongly agree’ response across these three items. Within this subdomain, 73.2% (*n* = 104/142) strongly agreed with item 5: ‘I teach because it’s important for me to make a contribution to students becoming a good healthcare professional in the future’. This also represented the highest percentage response out of all 18 items. Additionally, 42.3% (*n* = 60/142) and the most common response, strongly agreed with feeling like they had a sense of duty to pass on their knowledge to students (item 6).

A majority of participants strongly agreed with the ‘Intrinsic’ subdomain items 2 and 4, ‘I enjoy teaching most of the time’ and ‘I teach because it increases my job satisfaction’ respectively suggesting that altruism plays a strong role in why participants teach healthcare students.

Items 8 and 9 in the ‘Introjected’ subdomain demonstrated higher responses to the negative attitude Likert scale of agreements, supporting the notion that teaching is not typically driven by guilt.

The ‘Career’ subdomain exhibited the largest diversity of responses. For PTMQ ‘Career’ item 12, ‘I teach because it is good for my career progression,’ Medical Imaging participants strongly agreed (25%, *n* = 4/16) with this statement compared to Medicine 1.8% (*n* = 1/55) and Optometry 2.8% (*n* = 2/71). The negative attitude Likert responses to the same PTMQ career item, resulted in no Medical Imaging participants strongly disagreeing with this statement whereas 38.2% (*n* = 21/55) of Medicine and 25.4% (*n* = 18/71) of Optometry did. Teaching was viewed as a greater incentive for career progression in Medical Imaging compared to Medicine and Optometry.

In the ‘External’ subdomain, 65.6% (*n* = 93/142) of external healthcare educators strongly disagreed with the assertion that they taught to avoid being performance-managed (item 15). Items 13 and 14 also, most commonly, signalled that participants were not motivated to teach by the prospect of a reward or to avoid punishment.

Approximately 60% strongly disagreed with item 17, ‘I rarely feel like teaching but do it anyway’ and item 18, ‘I teach even though I often perceive it as an annoying chore’, indicating ‘Amotivation’ was not a prevalent trait amongst participants.

### PTMQ subdomain and item comparisons across Medicine, Optometry and Medical Imaging

Across the three professions (Medicine, Optometry and Medical Imaging), PTMQ participant responses showed a reasonable level of homogeneity (Table 3). The mean values for the ‘Intrinsic’ and ‘Identified’ items were consistently low, indicating that teaching motivations were primarily altruistic. In the ‘Introjected’ subdomain, the mean values suggest that feelings of guilt do not serve as a motivating factor for teaching across all professions.

Significant differences were observed within the ‘Career’ (*p* < 0.003) and ‘External’ (*p* < 0.001) subdomains. Specifically, item 12 in the ‘Career’ subdomain, “I teach because it is good for my career progression” and item 14 in the ‘External’ subdomain, “I mainly teach because it is part of my position description” highlighted this disparity, with Medical Imaging exhibiting a significantly lower mean value than Medicine and Optometry (*p* < 0.001).

The ‘Amotivation’ subdomain displayed similar higher mean values across all three professions, indicating there was no lack of motivation to teach. However, item 16, “I teach even though I feel that teaching is a lower priority than my other occupational activities”, revealed a significantly higher mean value for Medical Imaging compared to Medicine and Optometry (*p* < 0.001).

**Table 3:** PTMQ subdomain and item comparisons across Medicine, Medical Imaging and Optometry

Subdomain	Medicine	Medical Imaging	Optometry	*P* value
Intrinsic	1.87 ± 0.93	1.56 ± 0.17	1.74 ± 0.08	0.183
Identified	1.49 ± 0.08	1.40 ± 0.14	1.65 ± 0.07	0.146
Introjected	3.79 ± 0.14	3.28 ± 0.26	3.78 ± 0.13	0.200
Career	3.75 ± 0.13	2.19 ± 0.23	3.11 ± 0.11	0.003^a, b,c^
External	4.25 ± 0.11	3.21 ± 0.21	4.24 ± 0.10	0.001^a, c^
Amotivation	4.16 ± 0.12	4.42 ± 0.22	4.07 ± 0.11	0.368
**Survey Item**				
Item 1	1.94 ± 0.13	1.75 ± 0.23	1.92 ± 0.11	0.757
Item 2	1.68 ± 0.10	1.50 ± 0.18	1.56 ± 0.09	0.562
Item 3	2.00 ± 0.11	1.56 ± 0.21	1.94 ± 0.10	0.171
Item 4	1.83 ± 0.11	1.25 ± 0.20	1.58 ± 0.09	0.062
Item 5	1.22 ± 0.08	1.13 ± 0.14	1.45 ± 0.07	0.061
Item 6	1.80 ± 0.11	1.56 ± 0.21	1.83 ± 0.10	0.510
Item 7	1.44 ± 0.09	1.50 ± 0.16	1.66 ± 0.08	0.164
Item 8	3.46 ± 0.17	3.13 ± 0.30	3.68 ± 0.14	0.229
Item 9	4.11 ± 0.14	3.44 ± 0.26	3.89 ± 0.12	0.067
Item 10	4.00 ± 0.16	2.69 ± 0.29	3.49 ± 0.14	0.001^a, b,c^
Item 11	3.41 ± 0.16	1.81 ± 0.29	2.47 ± 0.14	0.001^a, b^
Item 12	3.85 ± 0.15	2.06 ± 0.27	3.37 ± 0.13	0.001^a, c^
Item 13	4.26 ± 0.14	3.00 ± 0.25	4.13 ± 0.12	0.001^a, c^
Item 14	3.89 ± 0.16	2.81 ± 0.29	4.01 ± 0.14	0.001^a, c^
Item 15	4.59 ± 0.10	3.81 ± 0.19	4.59 ± 0.09	0.001^a, c^
Item 16	3.70 ± 0.17	4.00 ± 0.32	3.55 ± 0.15	0.426^a, c^
Item 17	4.39 ± 0.12	4.56 ± 0.22	4.39 ± 0.11	0.772
Item 18	4.39 ± 0.13	4.27 ± 0.23	4.27 ± 0.11	0.260

Data represents means ± SEM. Superscript letters indicate significant Post hoc differences (*p* < 0.05) incorporating Bonferroni correction for multiple comparisons. ^a^ difference between Medical Imaging and Medicine; ^b^ difference between Medicine and Optometry; ^c^ difference between Optometry and Medical Imaging.

### PTMQ subdomain reliability

A good or acceptable Cronbach’s alpha was obtained for each motivational subdomain (Intrinsic α = 0.87, Identified α = 0.77, Introjected α = 0.86, Career α = 0.81, External α = 0.83 and Amotivation α = 0.83). Removal of a survey item within each subdomain did not increase Cronbach’s alpha except for in the Amotivation subdomain. By removing the first survey item in this subdomain, Cronbach’s alpha increased slightly from 0.83 to 0.85 however, we elected to keep the instrument intact rather than removing this question for a marginal gain.

### PTMQ factor analysis

The Kaiser-Meyer-Olkin measure of sampling adequacy was meritorious (0.804) [22].

Conducting the principal component analysis by fixing six domains as reported by Dybowski [16] resulted in similar outcomes to the initial validation. Bartlett’s test of sphericity was highly significant (c2 = 1334, df = 153, *p* < 0.001). Items had extraction coefficients between 0.685 and 0.873.

### PTMQ Rasch analysis

Following the classical factor analysis, a Rasch analysis was undertaken to further validate and confirm the results and to provide additional insight into the survey responses.

A significant likelihood ratio test result (χ^2^ = 148.87, df = 50, *p* < 0.0001) indicated that partial-credit parameterisation should be used [19]. Items 8–18 inclusive were reverse coded for the analysis. Overall, the data from the full PTMQ did not fit the Rasch model (Table 4), with a significant item-trait Chi-square interaction observed (χ^2^ = 80.45, df = 36, *p* < 0.0001).

**Table 4:** Rasch reliability and fit statistics for the full PTMQ, the two subgroups identified from multidimensionality assessment of the PTMQ, and each of the six individual subdomains in the PTMQ survey

				PTMQ Subgroups			PTMQ Subdomains					
	Parameters	Rasch Model Expectations	PTMQ	Altruistic^b^	Career^c^		Intrinsic	Identified	Introjected	Career	External	Amotivation
	Misfitting Items	Fit residual ± 2.5 logits^[**a**]^ [19]	None	-	-		None	None	None	None	None	None
Targeting	Item Location (logits)	Difference in person and item means < 1 [36]	0.00 ± 1.05	0.00 ± 1.21	0.00 ± 0.94		0.00 ± 1.06	0.00 ± 1.54	0.00 ± 1.93	0.00 ± 0.77	0.00 ± 1.60	0.00 ± 1.53
	Person Location (logits)		-1.46 ± 0.81	-2.57 ± 1.35	-1.07 ± 1.22		-3.17 ± 2.11	-5.47 ± 2.17	-2.34 ± 2.64	-0.50 ± 1.56	-2.79 ± 2.40	-2.60 ± 1.97
Item-trait Chi-square Interaction	χ2	-	80.45	67.41	35.59		12.48	3.94	2.46	8.49	3.13	7.44
	df	-	36	20	16		8	6	4	6	6	6
	p	*p* > 0.05^**a**^	< 0.0001	< 0.0001	0.003		0.13	0.69	0.65	0.19	0.79	0.28
	PSI	> 0.7 [37]	0.82	0.78	0.83		0.74	0.57	0.72	0.72	0.68	0.61
	Item fit residual (logits)	0 ± 1 [19]	0.23 ± 1.26	0.02 ± 2.23	0.20 ± 1.28		-0.15 ± 1.10	0.37 ± 0.51	0.24 ± 0.38	0.21 ± 0.71	0.23 ± 0.23	0.18 ± 0.57
	Person fit residual (logits)	0 ± 1 [19]	-0.20 ± 1.26	-0.18 ± 0.97	-0.25 ± 1.11		-0.40 ± 0.85	-0.20 ± 0.61	-0.34 ± 0.53	-0.40 ± 0.95	-0.23 ± 0.71	-0.36 ± 0.97
	PCA (Eigenvalue for 1st Contrast)	< 2.5 [2] [36]	4.93	2.31	2.53		1.84	1.64	1.95	1.66	1.60	1.95
	Variance by the first factor	> 50% [36]	27.40%	23.10%	31.70%		45.97%	57.75%	97.31%	55.30%	53.32%	64.93%
Differential Item Functioning (DIF)	Course	-	-	-	-		None	None	None	None	None	None
	Sex	-	-	-	-		None	None	None	None	Item 15 (F = 8.16, *p* = 0.0054)	None
	Teaching Years	-	-	-	-		None	None	None	None	None	None

a. Non-significant Chi-square values using a Bonferroni correction at the *p* = 0.01 level

b. Intrinsic, Identified, and Amotivation subdomains based on individual item loadings on PC1 from the PTMQ analysis

c. Introjected, Career, and External subdomains based on individual item loadings on PC1 from the PTMQ analysis

As the PTMQ was originally developed to target specific subdomains, the misfit of the full survey results to the Rasch model was assumed to be the result of multidimensionality in the instrument. This was confirmed by examining residual Principal Component 1 (PC1) (Table 4) [23]. Items 8–15 negatively correlated with PC1, dividing the questionnaire into two main groups (Intrinsic, Identified, and Amotivation subdomains in one ‘Altruistic’ group; Introjected, Career, and External subdomains in the other ‘Career’ group). No new subdomains were identified.

### PTMQ Rasch subgroup analysis

Rasch analysis was conducted on the ‘Altruistic’ and ‘Career’ subgroups identified from the PC1 loadings in the full PTMQ. Neither the Altruistic group (Intrinsic, Identified, and Amotivation subdomains; χ^2^ = 67.41, *p* < 0.0001) nor the Career group (Introjected, Career, and External subdomains; χ^2^ = 35.59, *p* = 0.003) fit the Rasch model (Table 4). (The principal component loading analyses for the two subgroups suggested, as the original PTMQ analyses found [16], that each of the six subdomains represents a different underlying trait of teacher motivation.

### PTMQ Rasch subdomain analysis

The six subdomains were analysed individually with Rasch to confirm they each represented a single underlying trait. All six subdomains fitted the Rasch model, albeit with reduced power of fit due to the smaller number of questions and thresholds (Table 4). All items in each subdomain showed good individual item fit residuals (within the expected ± 2.5 logit range) with no significant chi-square values. Many of the items showed disordered thresholds, but investigation of the category frequencies revealed this was mostly due to clustering of responses at one end of the Likert scale, and no collapsing of categories was required.

During the subdomain analysis, each item was examined for the presence of any differential item functioning (DIF) for each of the three person factors included in the model (course, sex, and year in teaching). Only item 15 displayed any DIF, showing a significant difference in item response by sex (F = 8.16, *p* = 0.005). Uniform DIF was present, suggesting that male tertiary educators in this cohort were more likely to indicate they taught to avoid being performance managed than expected, while female participants were less worried about this than expected in the model.

Differences in response (average person abilities) across the three person factors were also examined for each of the six subdomains (Table 5). Investigation of the mean person abilities showed that educators involved in Medical Imaging were more likely to score higher in the Career and External subdomains than those in Medicine or Optometry, indicating they were more likely to be teaching for the impact it would have on their career progression and the innate requirements of their position expectations.

**Table 5:** Person factor comparisons within each subdomain of the PTMQ

*Subdomain*			*Course*				*Sex*			*Time in Teaching*			
			**Medicine**	**Medical Imaging**	**Optometry**		**Male**	**Female**		**< 1 year**	**1–5 years**	**6–10 years**	**> 10 years**
**Intrinsic**	**Person Ability (logits)**		*-2.85 ± 2.07*	*-3.95 ± 1.82*	*-3.24 ± 2.17*		*-3.05 ± 1.95*	*-3.36 ± 2.33*		*-3.18 ± 2.32*	*-3.21 ± 2.08*	*-3.01 ± 2.11*	*-3.33 ± 2.20*
	**F-Statistic**		*1.79*				*0.74*			*0.12*			
	***p-value***		*0.17*				*0.39*			*0.95*			
**Identified**	**Person Ability (logits)**		*-5.67 ± 2.18*	*-6.10 ± 1.92*	*-5.17 ± 2.20*		*-5.36 ± 2.31*	*-5.64 ± 1.96*		*-5.25 ± 1.99*	*-5.38 ± 2.21*	*-5.48 ± 2.32*	*-5.88 ± 1.95*
	**F-Statistic**		*1.59*				*0.60*			*0.34*			
	***p-value***		*0.21*				*0.44*			*0.80*			
**Introjected**	**Person Ability (logits)**		*-2.45 ± 2.61*	*-1.08 ± 3.60*	*-2.54 ± 2.36*		*-2.35 ± 2.66*	*-2.34 ± 2.63*		*-2.03 ± 3.01*	*-2.17 ± 2.49*	*-2.71 ± 2.58*	*-2.42 ± 3.08*
	**F-Statistic**		*2.12*				*0.0003*			*0.41*			
	***p-value***		*0.12*				*0.99*			*0.75*			
**Career**	**Person Ability (logits)**		*-1.20 ± 1.30*	*1.12 ± 1.44*	*-0.32 ± 1.47*		*-0.53 ± 1.66*	*-0.46 ± 1.42*		*0.01 ± 2.14*	*-0.13 ± 1.30*	*-1.13 ± 1.47*	*-0.84 ± 1.80*
	**F-Statistic**		*18.17*				*0.07*			*4.48*			
	***p-value***		**< 0.0001**				*0.80*			**0.0049**			
**External**	**Person Ability (logits)**		*-3.01 ± 2.26*	*-0.53 ± 2.67*	*-3.12 ± 2.19*		*-2.97 ± 2.50*	*-2.51 ± 2.24*		*-2.18 ± 2.55*	*-2.80 ± 2.25*	*-3.48 ± 2.08*	*-1.85 ± 2.99*
	**F-Statistic**		*8.94*				*1.24*			*2.53*			
	***p-value***		**< 0.0001**				*0.27*			*0.059*			
**Amotivation**	**Person Ability (logits)**		*-2.66 ± 2.13*	*-3.02 ± 1.51*	*-2.46 ± 1.94*		*-2.27 ± 1.74*	*-3.09 ± 2.19*		*-1.72 ± 1.74*	*-2.58 ± 1.83*	*-2.56 ± 2.29*	*-3.22 ± 1.80*
	**F-Statistic**		*0.56*				*6.16*			*1.56*			
	***p-value***		*0.57*				**0.014**			*0.20*			

Significant values are presented in bold.

## Discussion

In Australia, the limited research exploring the motivations of external healthcare educators in teaching has predominantly centred around medical educators, with little attention given to other allied health professions. To the best of our knowledge, this is the first study in Australia to have applied a validated motivation questionnaire across different healthcare disciplines including Medicine, Optometry, and Medical Imaging professions. Our adaptation of Dybowski and Harendza’s PTMQ [16] did not compromise its application, demonstrating good internal consistency and construct validity.

Across all surveyed professions, altruism emerged as the overarching motivational factor behind why external healthcare educators teach in healthcare disciplines at Deakin University. These educators are motivated by a genuine desire to contribute to the development of future healthcare practitioners by imparting their skills and knowledge. This sense of altruism brings them significant internal satisfaction and fulfilment in their work, providing further motivation for them to continue teaching. These findings are comparable with global research, which commonly cited altruistic elements as healthcare educators’ primary reason for teaching [6]. Similarly, several Australian studies have also reported that clinical educators predominantly engage in teaching for the enjoyment it provides [11–13,15]. Interestingly, Thomson et al. [12], suggested that teaching institutions could benefit from marketing this altruistic enjoyment of teaching to recruit new educators. Adopting such a strategy could assist in addressing the growing need for external healthcare educators supervising students.

The majority of our healthcare educators perceived that they had a sense of duty to contribute to their profession and the future of the healthcare system. This finding is also consistent with research undertaken on Australian general practitioners, where 82% (*n* = 69) of participants felt the same ethical responsibility to teach [13]. As a result, it was not unexpected to find low levels of teaching amotivation within our study. While overall, there were low levels of amotivation, some gender differences were noted, with males slightly more likely to display amotivational tendencies than females.

Although, in general, there were no significant differences in participants’ motivational responses, some educators within the ‘Career and External’ subdomains exhibited additional driving factors independent of the more altruistic motivating aspects. Medical Imaging educators showed a greater inclination to agree with ‘Career and External’ motivation PTMQ items than Optometry and Medicine educators, indicating that they may be more driven to teach for career progression but also, display a greater level of indifference towards meeting others’ teaching expectations compared to Medicine and Optometry. This may be explained, in part, by the structure of the Medical Imaging workforce which is divided between hospital and non-hospital sectors. Many hospital-based positions build into their promotion policies minimum capability duties which, for some levels, include supervision and training of students, potentially increasing Medical Imaging educators’ incentive to mentor and teach [24]. The majority of the Medical and Optometric workforce surveyed are situated in private practice. Career progression often ceases in these settings, especially in smaller private practices therefore, the supervision and training of healthcare students tends not to be a strong factor for promotion.

To maintain maximum satisfaction and long-term commitment of external healthcare educators, gaining a better understanding of the perceived benefits and barriers associated with teaching could be invaluable. Currently, universities offer a wide variety of benefits and resources to incentivise external healthcare educators to teach. Several studies have found that the benefits perceived as being most valuable vary, but most commonly include the use of academic appointment titles, access to university libraries, and continued professional development (CPD) [25–27]. While our research found, overall, that the resources and benefits associated with teaching, including holding university email accounts, were not important to participants, having access to the university library was somewhat beneficial. Similarly, Scott and Sazegar [28] reported that medical educators regarded the provision of email accounts to be of little value; however, in contrast to our findings, their study indicated that library access was deemed unimportant.

Although all of our external healthcare educators are given academic appointment titles, our research did not investigate the perceived benefits of this. Considering, that other studies have shown this to be a major motivation to teach [25,26], it appears worthwhile for universities to continue to offer this benefit.

Continued professional development has also been recognised as an important teaching incentive [25,26]. Given that only a third of our participants engaged in university-provided continuing professional development opportunities, typically for five or fewer hours per year, there is potential for universities, including Deakin, to develop strategies that further incentivise participation. Baldor et al. [26], additionally found that CPD credits/points for teaching were rated highly important in medical educators’ decision to teach. Since most healthcare professionals/educators in Australia are required to undertake professional development to maintain their registration to practice, universities could consider a system that provides CPD points as a reward for their teaching hours.

There was a discernible difference in the perceived value of university assistance in research endeavours between Medicine and Optometry, with Medicine placing a higher value on this support compared to Optometry. This could be attributed to the fact that many external healthcare educators in Medicine are affiliated with hospitals that potentially include academic pursuits as part of their role whereas the vast majority of surveyed optometrists were based in community and private practice.

While this study revealed, overall, that there were no significant barriers to being a healthcare educator, the main deterrents influencing participants’ decision to be involved in teaching were competing work requirements and time constraints. This was more commonly cited by Medicine educators, than those in Optometry and Medical Imaging. Clinical supervision is widely known to be time-consuming and can negatively impact the number of patients seen in the practice [2,5,12,29]. Laurence et al. [29], imply the financial implications of this are more pronounced for the supervision of medical students compared to junior doctors and general practice registrars [29]. Kirkman et al. [2], also describe the demands of clinical practice and the responsibility of supervising optometry students to be higher in the early stages of a student’s placement compared to the later stages. This tension between patient care and clinical supervision lessens though as students’ skills improve. Reimbursement, in the form of a teaching stipend or subsidy rate to offset the cost of clinical teaching, has been proposed by several studies [27,29]. While this sounds like a reasonable solution, it may not be a financially viable option for many universities. Nonetheless, universities that continue to explore innovative solutions to address these challenges may help improve the retention rates of external healthcare educators.

Given that a majority of our participants teach without a paid appointment, it was unsurprising that this analysis demonstrated minimal financial barriers to teaching. Kirkman et al. [2], reported a similar outcome with clinical supervisors in Australian community-based optometric practices but also emphasised that financial incentives would be appreciated by external clinical supervisors to compensate them for their contributions - an aspect that this study did not explore.

Despite these apparent barriers, interest in clinical teaching continues to exist among external healthcare educators, likely driven by their altruistic motivations.

### Limitations

We acknowledge that there were several limitations in this study.

Part-one of the questionnaire did not utilise forced responses therefore, not all participants answered every question. This reduced the number of participants whose data could be analysed. However, several studies have shown that forcing responses to avoid missing data may compromise data quality as participants may not want to answer questions truthfully or may drop out before completing the survey [30,31].

The response rate may have generated a non-representative sample potentially affecting the validity of the results. Due to the convenience sampling methodology, those who responded are more likely to be engaged with, and theoretically motivated to contribute to teaching programs, potentially biasing the results. Our response rate was also lower than that reported by Dybowski and Harendza [16]. Some studies have suggested that falling survey response rates by medical practitioners can be attributed to workload and time pressures [32,33]. It is worth noting that during 2020, the COVID-19 pandemic had a profound negative impact on the workload and stress levels of Australian healthcare workers [34,35], which possibly contributed to the lower-than-expected response rate.

This single-centred study involved only three healthcare professions, potentially limiting the generalisability of the results. Mitigating this possibility is the fact that all participants are based in geographically disparate areas and from different professions (medical and allied health). We also acknowledge that there may be an institutional bias and that external healthcare educators associated with other universities may respond differently from the participants of this study. Despite a relative overrepresentation of Optometry educators and a relative underrepresentation of Medical Imaging educators, the vast majority of responses exhibited consistent patterns across all professions, again speaking to the generalisability of the results. Nonetheless, even with a smaller number of Medical Imaging participants responding, subtle yet significant differences in teaching motivations between the professions’ were still discernible.

### Future research

To validate our findings, broadening the application of the PTMQ to other healthcare disciplines and institutions not targeted in this study would allow for a more diverse sample and greater comparability of teaching motivations amongst external healthcare educators. This could potentially provide additional insights into how universities can tailor their curriculum and incentives to better maintain retention rates among external healthcare educators. Moreover, this study did not capture any information on the geographical location of the participating external healthcare educators. This information would be beneficial because it is vital for rural and regional training programs, such as those at Deakin University, to maximise its external staff uptake in the hopes that graduating students bolster the workforce in these areas. Another worthwhile consideration would be to organise a follow-up focus group discussion on the same sample to further explore the issues raised, such as competing work requirements and time constraints and map any divergence of findings.

## Conclusion

This study has demonstrated that the PTMQ exhibits good generalisability when applied across Australian medical and allied health professions. Altruism was the overarching motivational theme for why external healthcare educators contribute to university healthcare programs. However, career motivations differed slightly across the professions, with Medical Imaging educators showing a stronger inclination towards teaching for the promotional benefits associated with their professional award. While there were no key benefits or barriers identified in maintaining teaching relationships with the university, exploring innovative ideas to address time constraints faced by some external healthcare educators is essential to enhancing teaching participation. Moreover, given the rising demand for clinical placements, external healthcare educators will remain pivotal in the provision of authentic clinical experiences and professional development opportunities for healthcare students. Consequently, universities must remain cognisant of the factors that aid in the recruitment and retention of their external healthcare educators and continue to maximise these for the ongoing success and sustainability of their clinical programs.

### Electronic supplementary material

Below is the link to the electronic supplementary material.

Supplementary Material 1

## Data Availability

All data generated and/or analysed during this study are included in this published article [and its supplementary information files].

## Abbreviations

DIF: Differential Item Functioning
HR: Human Resources
IT: Information and Technology
PC1: Principal Component 1
PC2: Principal Component 2
PTMQ: Physician Teaching Motivation Questionnaire
SD: Standard Deviation
SDT: Self Determination Theory
