# Supplementary material for: Understanding barriers, enablers and motivational factors for Australian healthcare educators teaching university students on clinical placement using the validated Physician Teaching Motivation Questionnaire
Source: BMC Med Educ. 2024 Aug 21;24:900. doi: 10.1186/s12909-024-05886-1 (PMC11337651; doi:10.1186/s12909-024-05886-1)
Supplement: Supplementary file 1 — Supplementary Material 1 [file 12909_2024_5886_MOESM1_ESM.docx]

Teaching Motivation Questionnaire:

Part One and Part Two

**Thank you for considering assisting the Deakin University School of Medicine to better understand the contribution of our valued external teachers. This survey seeks to better understand what aspects of the teaching role you value the most in order to improve the teaching and learning experience for both you and our students.**
 

**Administration of this questionnaire has been granted approval by the Deakin University Faculty of Health Human Ethics committee HEAG-H_184-2020.**
 
**All your responses are completely anonymous. Your level of participation and response to questions will not influence your relationship with the University in any way. The researchers thank you in advance for completing this survey. Should you choose not to participate simply close this browser window.**
 
 
Click here for Plain Language Statement

**By clicking the next arrow below and completing the survey your consent to participate is implied.**

Q1 Which of the following options **best** describes the title of your Deakin University appointment?

- Casual
- Honorary
- Adjunct
- Visitor
- Conjoint

Q2 How long have you held this appointment at Deakin University?

- Less than 1 year
- 1-5 years
- 5-10 years
- >10 years

Q3 Is this a paid appointment?

- Yes
- No

Q4 Do you hold an appointment (conjoint, honorary or otherwise) with any other university?

- Yes
- No

Display This Question:

If Do you hold an appointment (conjoint, honorary or otherwise) with any other university? = Yes

Q5 How long have you held an appointment with another university?

- Less than 1 year
- 1-5 years
- 5-10 years
- >10 years

Q6 What is your age group?

- 18-29 years
- 30-39 years
- 40-49 years
- 50-59 years
- 60-69 years
- over 70 years

Q7 What is your gender?

- Male
- Female
- Trans/Gender diverse
- Prefer not to say

Q8 What is your highest educational qualification?

- Bachelors degree
- Post Graduate Diploma
- Masters degree
- Doctor of Philosophy (please specify main topic of PhD) __________________________________________________

Q9 Do you hold a Fellowship with any college?

- Yes
- No

Q10 Which Deakin students do you teach? (click all that apply)

- Medicine
- Medical Imaging
- Optometry
- Other, please specify __________________________________________________

Q11 How would you describe your teaching activities for Deakin? (click all that apply)

- Delivering lectures
- Delivering tutorials
- Clinical skills teaching
- Research training
- Supervising students providing clinical care

Q12 In which location do you teach Deakin University students? (click all that apply)

- Hospital
- Australian College of Optometry
- Community Health Centre
- Group Practice
- Solo Practice
- Deakin Campus

**The following questions seek to better understand your perceptions of services that the University may offer you.**

Q13 How important is having a Deakin email account to you?

- Extremely important
- Very important
- Moderately important
- Slightly important
- Not at all important

Q14 How often do you use your email account?

- Daily
- Weekly
- A few times a month
- A few times a year
- Never

Q15 How important is having access to the Deakin library to you?

- Extremely important
- Very important
- Moderately important
- Slightly important
- Not at all important

Q16 How often do you access the Deakin library?

- Daily
- Weekly
- A few times a month
- A few times a year
- Never

Q17 How important is it to you that Deakin can assist you with your research endeavours?

- Extremely important
- Very important
- Moderately important
- Slightly important
- Not at all important

Q18 Are there any barriers to your association with Deakin? (click all that apply)

- No barriers
- The requirements of the HR onboarding process
- The requirements of ongoing compliance i.e. course meetings / course paperwork / workplace safety training
- Conflict of Interest
- Competing work requirements / time management
- Income expectations
- IT requirements
- Other __________________________________________________

Q19 How important is it that you have access to the online teaching material (e.g. lectures, clinical skills practicals) provided to Deakin students?

- Extremely important
- Very important
- Moderately important
- Slightly important
- Not at all important

Q20 How often do you access online teaching material provided to Deakin students?

- Daily
- Multiple times a week
- Multiple times per semester/ trimester/ rotation
- Once per semester/ trimester/ rotation
- Never

Q21 Do you access professional development events (workshops / training) run by Deakin to assist you in developing your teaching skills?

- Yes
- No

Skip To: Q22 If Do you access professional development events (workshops / training) run by Deakin to assist you... = Yes

Q22 How many hours of professional development do you access through Deakin per year?

- Nil
- 1-5
- 6-20
- > 20

Display This Question:

If Is this a paid appointment? = Yes

Q23 Does the University remunerate you for this professional development?

- Yes
- No

Q24 How would you describe your teaching activities for Deakin? (click all that apply)

- Deliver timetabled lecture(s)
- Deliver timetabled tutorial(s)
- Deliver timetabled clinical skills training in laboratory space
- Supervising students on clinical placement
- Research training
- Non-timetabled informal tutorials at the clinic

Display This Question:

If Which Deakin students do you teach (click all that apply)? = Medicine

Q25 Are you paid for you teaching role at Deakin separately from designated clinical support time allocated by your employer?

- Yes
- No

Display This Question:

If Are you paid for you teaching role at Deakin separately from designated clinical support time all... = Yes

Q26 How many hours of paid teaching do you perform on average, each week?

- less than 2
- between 2 and 10
- more than 10

| Page Break |  |
| --- | --- |

**The following questions are drawn from a validated survey that seeks to better understand your motivation for teaching. Please choose the response that best describes your reaction to each question.**

Question 1

|  | Strongly agree | Somewhat agree | Neither agree nor disagree | Somewhat disagree | Strongly disagree |
| --- | --- | --- | --- | --- | --- |
| I look forward to my next teaching session most of the time |  |  |  |  |  |

Question 2

|  | Strongly agree | Somewhat agree | Neither agree nor disagree | Somewhat disagree | Strongly disagree |
| --- | --- | --- | --- | --- | --- |
| I enjoy my teaching most of the time |  |  |  |  |  |

Question 3

|  | Strongly agree | Somewhat agree | Neither agree nor disagree | Somewhat disagree | Strongly disagree |
| --- | --- | --- | --- | --- | --- |
| I am completely in my element when teaching |  |  |  |  |  |

Question 4

|  | Strongly agree | Somewhat agree | Neither agree nor disagree | Somewhat disagree | Strongly disagree |
| --- | --- | --- | --- | --- | --- |
| I teach because it increases my job satisfaction |  |  |  |  |  |

Question 5

|  | Strongly agree | Somewhat agree | Neither agree nor disagree | Somewhat disagree | Strongly disagree |
| --- | --- | --- | --- | --- | --- |
| I teach because it’s important for me to make a contribution to students becoming good healthcare professionals in the future |  |  |  |  |  |

Question 6

|  | Strongly agree | Somewhat agree | Neither agree nor disagree | Somewhat disagree | Strongly disagree |
| --- | --- | --- | --- | --- | --- |
| I teach because I am convinced it’s my duty to pass on my knowledge |  |  |  |  |  |

Question 7

|  | Strongly agree | Somewhat agree | Neither agree nor disagree | Somewhat disagree | Strongly disagree |
| --- | --- | --- | --- | --- | --- |
| I teach because I feel that the knowledge I impart is important |  |  |  |  |  |

Question 8

|  | Strongly agree | Somewhat agree | Neither agree nor disagree | Somewhat disagree | Strongly disagree |
| --- | --- | --- | --- | --- | --- |
| I teach because otherwise I feel guilty for not helping my colleagues |  |  |  |  |  |

Question 9

|  | Strongly agree | Somewhat agree | Neither agree nor disagree | Somewhat disagree | Strongly disagree |
| --- | --- | --- | --- | --- | --- |
| I teach because otherwise I feel guilty for not helping my supervisors |  |  |  |  |  |

Question 10

|  | Strongly agree | Somewhat agree | Neither agree nor disagree | Somewhat disagree | Strongly disagree |
| --- | --- | --- | --- | --- | --- |
| I teach because it is good for my CV to accomplish my occupational objectives |  |  |  |  |  |

Question 11

|  | Strongly agree | Somewhat agree | Neither agree nor disagree | Somewhat disagree | Strongly disagree |
| --- | --- | --- | --- | --- | --- |
| I teach because it is advantageous to my occupation |  |  |  |  |  |

Question 12

|  | Strongly agree | Somewhat agree | Neither agree nor disagree | Somewhat disagree | Strongly disagree |
| --- | --- | --- | --- | --- | --- |
| I teach because it’s good for my career progression |  |  |  |  |  |

Question 13

|  | Strongly agree | Somewhat agree | Neither agree nor disagree | Somewhat disagree | Strongly disagree |
| --- | --- | --- | --- | --- | --- |
| I teach most of the time because my supervisors expect it from me |  |  |  |  |  |

Question 14

|  | Strongly agree | Somewhat agree | Neither agree nor disagree | Somewhat disagree | Strongly disagree |
| --- | --- | --- | --- | --- | --- |
| I mainly teach because it is part of my position description |  |  |  |  |  |

Question 15

|  | Strongly agree | Somewhat agree | Neither agree nor disagree | Somewhat disagree | Strongly disagree |
| --- | --- | --- | --- | --- | --- |
| I mainly teach because otherwise I could be performance managed |  |  |  |  |  |

Question 16

|  | Strongly agree | Somewhat agree | Neither agree nor disagree | Somewhat disagree | Strongly disagree |
| --- | --- | --- | --- | --- | --- |
| I teach even though I feel that teaching is a lower priority than my other occupational activities |  |  |  |  |  |

Question 17

|  | Strongly agree | Somewhat agree | Neither agree nor disagree | Somewhat disagree | Strongly disagree |
| --- | --- | --- | --- | --- | --- |
| I rarely feel like teaching but do it anyway |  |  |  |  |  |

Question 18

|  | Strongly agree | Somewhat agree | Neither agree nor disagree | Somewhat disagree | Strongly disagree |
| --- | --- | --- | --- | --- | --- |
| I teach even though I often perceive it as an annoying chore |  |  |  |  |  |

|  |  |
| --- | --- |

**Thank you for completing this questionnaire and for your valued contribution to the Deakin University School of Medicine.**
